# Pylluminator: fast and scalable analysis of DNA methylation data in Python

**DOI:** 10.1093/bioadv/vbag146

**Published:** 2026-05-26

**Authors:** Elio Fanchon, Benjamin Loire, Jean-Philippe Trani, Frédérique Magdinier, Anaïs Baudot

**Affiliations:** Aix-Marseille Univ, INSERM, MMG, Marseille, France; Aix-Marseille Univ, INSERM, MMG, Marseille, France; Neurology Therapeutic Area, R&D Servier Paris-Saclay Institut, Gif-sur-Yvette, France; Aix-Marseille Univ, INSERM, MMG, Marseille, France; Aix-Marseille Univ, INSERM, MMG, Marseille, France; Aix-Marseille Univ, INSERM, MMG, Marseille, France; CNRS, Marseille, France

## Abstract

**Motivation:**

Illumina Infinium BeadChip technology for DNA methylation analysis continues to expand, with the latest EPICv2 arrays targeting about a million loci. As data volumes from this technology continue to grow, there is increasing demand for more scalable data processing solutions. Meanwhile, Python has gained significant interest in bioinformatics for its efficiency, versatility, and widespread use in data science and machine learning. Yet, no comprehensive Python toolkit exists for Illumina methylation array analysis.

**Results:**

We present Pylluminator, a Python implementation of essential analysis methods including pre-processing tools, quality control, differential methylation analysis, and visualizations. Based on the established R packages SeSAMe and ChAMP, Pylluminator provides a scalable, user-friendly toolkit for DNA methylation analysis.

**Availability and implementation:**

Pylluminator is an open-source package under MIT license available at https://github.com/eliopato/pylluminator. It was developed using Python 3.12 and can be installed with pip and uv. The documentation with thorough installation instructions and examples can be found at https://pylluminator.readthedocs.io

## 1 Introduction

Epigenetics, a rapidly expanding field in biology, refers to the study of heritable changes able to modify the genome activity without alteration of the DNA sequence itself. Epigenetics involves chemical modifications of the DNA molecule, modifications of histone proteins around which DNA is wrapped to form chromatin, and the regulatory action of non-coding RNAs (Li 2021).

DNA methylation, one of the key epigenetic chemical modifications of DNA, is a powerful genome regulation mechanism. It plays a causative role in the development of various pathologies, including cancer (Sharma *et al.* 2010), autoimmune disorders, neurological disorders or rare genetic diseases such as ICF (Immunodeficiency, centromeric instability and facial anomalies), or Tatton-Brown-Rahman syndromes among others (Moosavi and Ardekani 2016). DNA methylation occurs when a methyl group is added to the DNA molecule on an adenine or cytosine base (Ratel *et al.* 2006). In mammals, DNA methylation mainly occurs on the cytosines of CpG dinucleotides and the majority of CpG dinucleotides (60%–90%) are methylated. CpG-rich regions called ’CpG islands’, which are frequently found in gene promoter regions, show variable methylation patterns (Bird 1986). Methylation of CpG islands usually contribute to gene silencing. Methylation of repetitive DNA sequences also contribute to the maintenance of genome integrity.

Illumina Infinium Methylation BeadChips offer high-resolution tools to quantify genome-wide DNA methylation (Bibikova *et al.* 2006). Various bioinformatics packages have been developed over the years to process and analyze Illumina Infinium BeadChips data: ChAMP (Tian *et al.* 2017), SeSAMe (Triche *et al.* 2013) or Minfi (Aryee *et al.* 2014) in R, or Methylsuite [methylprep], CpGTools (Wei *et al.* 2021) or Mepylome (Brugger *et al.* 2026) in Python. Unfortunately, the R packages are being challenged by the increasing number of probes and samples in experiments. For instance, the latest Illumina Infinium BeadChips version for the human genome, EPICv2, targets over 930.000 loci, of which 99.5% are CpG sites. The Python packages are either not maintained (Methylsuite) or lacking pre-processing and analysis features (CpGTools, Mepylome).

**Figure 1:**
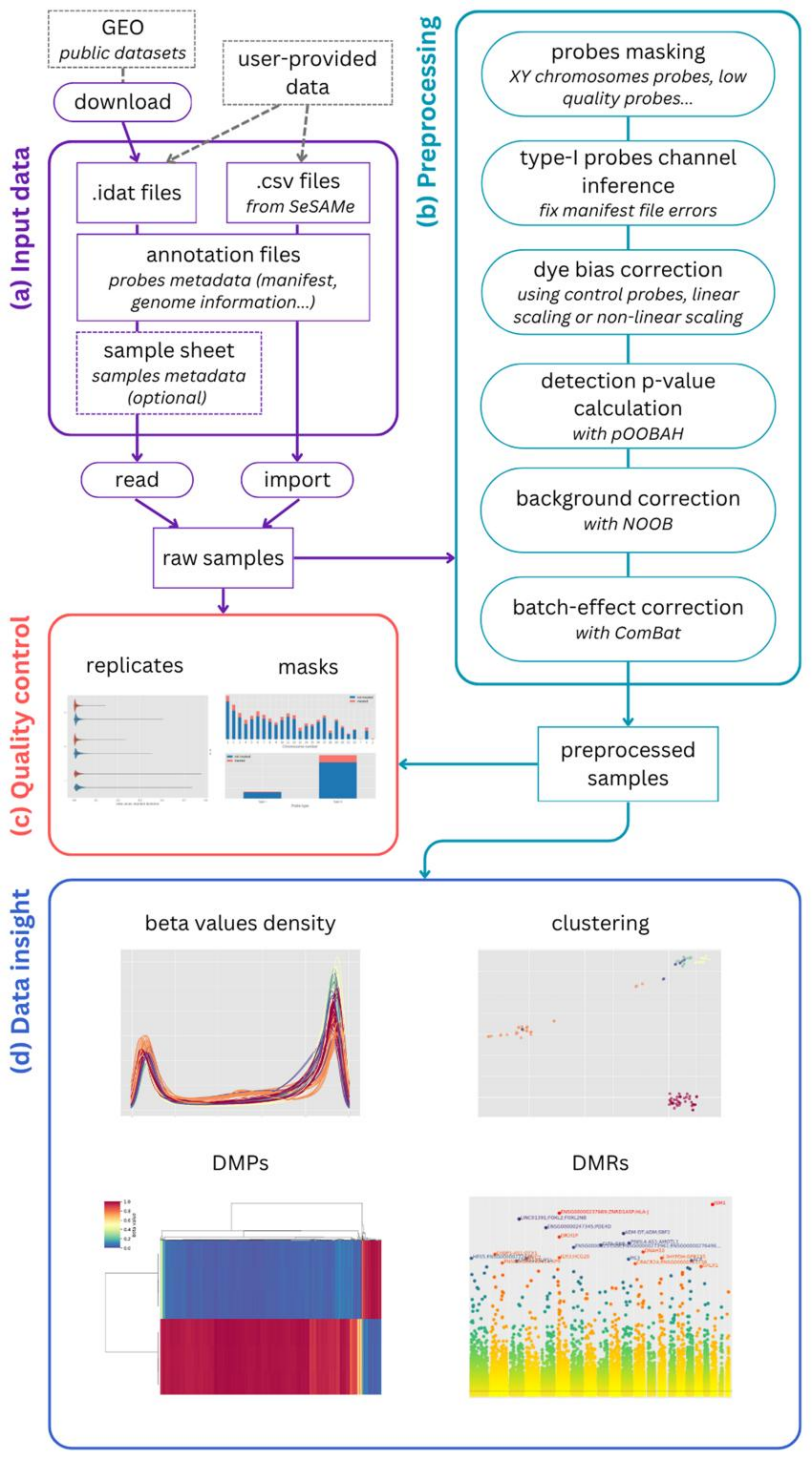
Flowchart of the main features of Pylluminator.

With the exponential growth of interest in data science over the past decade, Python became one of the most used languages to handle large-scale datasets efficiently. An increasing number of bioinformatics tools are being developed or translated to Python, but there is no up-to-date tool available to fully process, analyze, and visualize Illumina methylation array data. We propose to fill the gap with Pylluminator, which implements the most common pre-processing methods, analysis algorithms, and data visualizations of DNA methylation (Fig. 1). Pylluminator is based on the reference R packages SeSAMe and ChAMP and provides a user-friendly, scalable, and reproducible toolkit for methylation data analysis, bridging the gap between bioinformatics and data science. Where a function also exists in SeSAMe, it is designed and tested to match its output and replicate its options.

## 2 Input data

Pylluminator handles all human (27k, 450k, MSA, EPIC, EPIC+, EPICv2), mammalian (Mammal40) and mouse (MM285) array versions, and supports different genome versions (hg38 and hg19 for human arrays, mm39 and mm10 for mouse arrays).

The annotations corresponding to each array, which we have created from SeSAMe and Illumina annotation files, are automatically downloaded from https://github.com/eliopato/pylluminator-data when needed—but custom annotations can also be provided by the users. The annotation files include the manifest file that maps each probe’s Illumina ID to its metadata (e.g., color channel, location on the chromosome, probe type). The annotation files also include genome information files for data enrichment and visualizations (e.g., transcripts description, chromosome regions information).

As described in Fig. 1(a), the input methylation data can be raw.*idat* files, as well as an existing SigDf object exported from SeSAMe as a.*csv* file. The raw.*idat* files can come from user-provided experimental data, or can be automatically downloaded from Gene Expression Omnibus (GEO) database by providing the samples GSM ids.

## 3 Pre-processing

As recommended by SeSAMe, the pre-processing pipeline starts by masking unwanted probes (Fig. 1(b)). Unwanted probes can be control probes, SNP probes, all non-CG probes, non-unique probes, probes that are known to have design issues (as defined by Zhou *et al.* [2017]), XY chromosome probes, as well as user-defined masks. The masking framework in Pylluminator makes it easy to exclude probes from the study, and masks are reversible at any time in the pipeline.

The second pre-processing step aims to infer the channel of type I probes. Type I and type II probes differ in design: type I probes use separate probes for methylated and unmethylated states, each measured in a distinct color channel, whereas type II probes use a single probe to measure both states in the same channel. As detailed in the ’Input Data’ section, probes metadata, including color channel for type I probes, are provided by the manifest files. Running the Type-I channel inference method before any further processing allows to correct potential errors in the annotation of type I probes.

Then, dye bias, a well-known issue in multichannel microarray experiments that is due to a difference in fluorescence intensity between the green (Cy3) and red (Cy5) channels, can be corrected with linear or nonlinear scaling.

Next, detection *P*value are commonly used to assess the likelihood that a given probe signal corresponds to background fluorescence. We chose to integrate pOOBAH (*P*value with Out-Of-Band Array Hybridization) (Zhou *et al.* 2018), a method that calculates detection *P*value based on the empirical cumulative distribution function of overall Type-I out-of-band signal. Probes with detection *P*value higher than a certain threshold are then masked (by default, 0.05). Zhou *et al.* (2018) also show that masking these probes can highly reduce technical variations, such as batch effect (Leek *et al.* 2010).

For overall background signal correction, Pylluminator implements NOOB, the background subtraction algorithm based on Normal-exponential deconvolution using Out-Of-Band probes, as defined by Ritchie *et al.* (2007) and Triche *et al.* (2013).

If a batch effect remains, Pylluminator also integrates the most widely used algorithm for correcting technical biases in microarray data, ComBat (Johnson *et al.* 2007). ComBat is based on an empirical Bayes framework, and handles low sample sizes and more than two batches at the same time.

## 4 Samples quality control

Pylluminator includes comprehensive quality control (QC) tools to identify potential issues in methylation data. Users can evaluate key metrics such as probe detection rates by probe type (CG, CH, SNP), average signal intensity by probe design type (I or II) and channel (red or green), type I probes color channel balance, and beta values distribution by probe type. These tools can be used to assess the quality of raw input data and the effectiveness of the pre-processing pipeline.

To further diagnose technical variability, the replicate analysis plot helps detect batch effects by comparing technical replicates. Additionally, visualizing the distribution of masked probes per chromosome and by probe design type provides valuable insights into experiment quality (Fig. 1 (c)).

## 5 Data insights

Beta-values are calculated for each probe using SeSAMe’s formula, defined as:


(1) $$\beta_{i}=\frac{\max(y_{i,\mathrm{methy}},1)}{\max(y_{i,\mathrm{methy}}+y_{i,\mathrm{unmethy}},2)}$$

where $y_{i,\mathrm{methy}}$ and $y_{i,\mathrm{unmethy}}$ are the intensities measured by the $i^{th}$ methylated and unmethylated probes, respectively.

The beta values density plot function offers options to plot each sample separately, or to group samples based on metadata (e.g. controls vs patients, or per batch to control for batch effect). Beta values of probes linked to a specific gene can be visualized in a heatmap with hierarchical clustering of samples, alongside the genomic positions of the probes.

Pylluminator provides an interface to the scikit-learn (Pedregosa *et al.* 2011) dimension reduction library, offering 14 different methods for unsupervised clustering of the samples, including PCA, MDS and FA. In addition to computing embeddings, it also generates visualizations to facilitate the interpretation of sample clustering patterns (Fig. 1(d)). The dendrogram visualization also gives information on the similarity between samples.

To detect Differentially Methylated Probes (DMPs), we fit an Ordinary Least Squares (OLS) linear regression model to each probe’s methylation values. By default, the model uses the M-values (logit-transformed beta values) as the dependent variable, as they are more statistically appropriate than beta values for linear regression due to their homoscedasticity and unbounded range. However, we provided the option to use beta-values, for consistency with SeSAMe. Any metadata extracted from the sample sheet (e.g., age, sex, phenotype) can be used as predictor in the formula to describe the model. The resulting *P*value and effect size are then used to identify the significant DMPs, after correcting for multiple testing using the Benjamini-Hochberg procedure (Benjamini and Hochberg 1995). If the experimental design includes technical replicates, repeated measures, or any other source of random variability, a Linear Mixed Model (LMM) is used instead of OLS. In the LMM, predictors of direct interest (e.g., age, sex, phenotype) are modeled as fixed effects, while sources of random variability (e.g., patient ID to account for repeated measures, batch ID) are modeled as random effects. This accounts for within-group correlations and improves the accuracy of inference. The identified DMPs can be visualized in a heatmap, with flexible options to select probes based on effect size or *P*value, to display sample metadata, and to incorporate hierarchical clustering for both samples and probes (Fig. 1(d)).

The method implemented to compute the Differentially Methylated Regions (DMRs) from DMPs is the same as in SeSAMe. Neighboring probes with similar methylation patterns for a given predictor contrast (e.g., treated vs. control) are grouped. Similarity is calculated based on the Euclidean distance between probes’ beta values. The distance cutoff used to separate the regions can be explicitly specified, or automatically set to the value at a specified quantile of all pairwise distances (by default, 0.5, i.e., the median). The significance of each DMR is then computed by combining the *P*value of its constituent DMPs with Stouffer’s *Z*-score method (Stouffer *et al.* 1949). A Manhattan plot annotated with gene information can be generated from the DMRs (Fig. 1(d)).

The Copy Number Variation (CNV) calculation method implemented in Pylluminator relies on copy-number-normal samples to normalize probe signal intensity. By default, Pylluminator provides precomputed normal reference data for EPIC/hg38 and EPICv2/hg38 array versions, similar to SeSAMe’s implementation. For other array versions, users must provide their own reference data. Regardless of the array version, it is strongly recommended to use normal samples that closely match the biological and technical characteristics of the target samples (e.g., same tissue type, platform, and processing batch) to ensure accurate normalization.

For Copy Number Segmentation (CNS), Pylluminator employs the Circular Binary Segmentation (CBS) algorithm to identify genomic regions with homogeneous CNV. This approach efficiently detects breakpoints and segments the genome into intervals of consistent copy number variation. The resulting segments and CNV values can be visualized in a Manhattan plot.

Pathway analysis methods provide valuable insights into the biological processes affected by differentially expressed genes (DEGs). To facilitate this, pylluminator offers a streamlined interface to the GSEApy package, enabling both Over-Representation Analysis (ORA) and Gene Set Enrichment Analysis (GSEA).

## 6 Results

We compared the major data processing and analysis functions of Pylluminator with their SeSAMe or ChAMP equivalents on a dataset of 24 EPIC samples from Laberthonnière *et al.* (2023). Computation time is evaluated by running the functions 10 times, on a laptop with 32 Gb RAM and an Intel Core i7 processor with 14 cores. Batch correction is applied using Sentrix ID as batch identifier, and samples’ phenotype as covariate. We compute the DMPs using the samples phenotype predictor (Control/FSHD1/FSDH2/BAMS). Results are presented in Table 1.

**Table 1:** Comparison of average execution time ± standard deviation (in seconds, unless otherwise stated) of the main functions.

Function	SeSAMe v1.24.0	ChAMP v2.36.0	Pylluminator v1.0
Read samples	51.42±3.66	40.40±1.39^a^	16.79±1.10
Beta values calculation	5.96±1.68		0.07±0.00
Non-linear dye bias correction	21.62±1.05	–	5.78±0.24
pOOBAH	12.14±0.73	–	2.04±0.07
NOOB	19.92±3.43	–	2.51±0.11
ComBat	–	18.67±0.56	5.37±0.16
DMPs	1130.52±38.62	113.76±2.35	127.43±1.55
DMRs	21.53±0.48	61.52±1.75	25.00±0.31
CNV on all samples^b^	–	49*min*	13.99±0.45
CNV + CNS on one sample^c^	9.29±0.41	–	6.35±0.40

a The champ.load() function includes the beta values calculation.

b We use here ChAMP’s default setting which is Copy Number Variation (CNV) calculation on all samples, without Copy Number Segmentation (CNS).

c We use here SeSAMe’s default setting which is CNV calculation on one sample, followed by Copy Number Segmentation (CNS).

## Data Availability

The data used in this article for the speed tests are available in the NCBI Gene Expression Omnibus (https://www.ncbi.nlm.nih.gov/geo/) with the access number GSE175527. Pylluminator’s code and data are hosted on GitHub and are publicly available at https://github.com/eliopato/pylluminator and https://github.com/eliopato/pylluminator-data.

## Code reliability

The code follows PEP8 standards and comes with extensive documentation, making it easy for external contributors to extend Pylluminator’s functionalities.

To ensure consistency and reliability, 93% of the code is covered by unit tests that systematically check for regressions or unwanted changes, and compare the results of Pylluminator with the outputs of reference tools such as SeSAMe (Triche *et al.* 2013) and ChAMP (Tian *et al.* 2017).

As Pylluminator is implemented as a fully independent and standalone tool, updates to external packages will not affect its functionality. New functions can be readily integrated thanks to this modularity and the existing testing infrastructure.
